# Structural and Functional Characterization of LIMCH1 and Its Agmatinase-like Region: A Case of Catalysis in a Highly Disordered Protein

**DOI:** 10.3390/biom15111620

**Published:** 2025-11-18

**Authors:** María-Belén Reyes, Allison Fuentes, Diego Bustamante, Fernando Retamal, Ignacia Lillo, Cristián Villegas, Juan-Pablo Carrasco, Martin Pereira-Silva, Marcell Gatica, Juan Román, Maximiliano Figueroa, Yamil Neira, José Martínez-Oyanedel, Víctor Castro-Fernández, Elena Uribe

**Affiliations:** 1 Departamento de Bioquímica y Biología Molecular, Facultad de Ciencias Biológicas, Universidad de Concepción, Concepción 4070409, Chile; marireyesc@udec.cl (M.-B.R.); allifuentes2016@udec.cl (A.F.); dbustamant2016@udec.cl (D.B.); feretamal2020@udec.cl (F.R.); iglillo2025@udec.cl (I.L.); crivillegas@udec.cl (C.V.); jcarrasco2019@udec.cl (J.-P.C.); marcgatica@udec.cl (M.G.); jroman@udec.cl (J.R.); maxifigueroa@udec.cl (M.F.); jmartine@udec.cl (J.M.-O.); 2 Departamento de Biología, Facultad de Ciencias, Universidad de Chile, Ñuñoa, Santiago 7800003, Chile; martin.pereira@ug.uchile.cl (M.P.-S.); vcasfe@uchile.cl (V.C.-F.); 3 Departamento de Análisis Instrumental, Facultad de Farmacia, Universidad de Concepción, Concepción 4030000, Chile; yneira@udec.cl

**Keywords:** LIMCH1, circular dichroism spectroscopy, agmatine, agmatinase

## Abstract

Agmatine is a biogenic amine that functions as a neurotransmitter and exhibits anticonvulsant, antineurotoxic, and antidepressant properties. It can be metabolized into putrescine and urea by canonical agmatinases or by the agmatinase-like protein (ALP), which corresponds to the C-terminal region of the LIMCH1 protein. The amino acid sequence of ALP/LIMCH1 diverges significantly from that of canonical agmatinases and lacks the conserved residues typically required for coordination with Mn^2+^, an essential cofactor for ureohydrolase activity. The three-dimensional structure of ALP/LIMCH1 remains unresolved, and predictive artificial intelligence algorithms such as AlphaFold have failed to model it reliably. As a result, the configuration of its active site and the identity of potential metal-coordinating ligands remain elusive. In this study, we purified recombinant full-length rat LIMCH1 (119.5 kDa) and a truncated ALP variant, ΔLIM-ALP (51 kDa), and analyzed their secondary structures using circular dichroism spectroscopy. Our results indicate that both proteins differ markedly from known ureohydrolases, exhibiting a high proportion of disordered regions (~60%) and β-structures (~30%). In contrast, *Escherichia coli* agmatinase displays a well-defined α/β/α sandwich fold. Despite these structural differences, ALP/LIMCH1 remain the only known mammalian proteins exhibiting agmatinase activity. To gain insight into the putative active site of ALP, we proposed candidate Mn^2+^-binding residues and generated single-point mutants (N213A, Q215A, D217A, E288A, K290A). Although these mutations did not significantly alter Mn^2+^ binding or its overall content in the protein samples, four mutants exhibited a decreased *K_m_* for agmatine and a reduced *V_max_* when normalized to protein concentration.

## 1. Introduction

Agmatine (1-amino-4-guanidinobutane) is a biogenic amine produced through the decarboxylation of arginine in a reaction catalyzed by arginine decarboxylase (ADC). While ADC is well-characterized in prokaryotes, its presence and function in mammals remain uncertain. It has been postulated that the primary source of agmatine in mammals is the gut microbiota [1].

Agmatine has been associated with a broad range of physiological and pharmacological effects in mammals, including multi-receptor binding and significant therapeutic potential [2,3,4]. Various studies have reported its beneficial actions, such as antinociceptive [5], anxiolytic [6], neuroprotective [7,8], antidepressant [9,10], anticonvulsant [11,12], and anti-inflammatory effects [13,14], as well as enhancement of working memory [15]. Preclinical studies have demonstrated that agmatine administration may be beneficial in conditions such as depression, anxiety, hypoxic–ischemic injury, pain perception, morphine tolerance, memory impairment, Parkinson’s disease, Alzheimer’s disease, traumatic brain injury, and epilepsy [14,15].

Interestingly, metformin, a widely used antidiabetic drug, was recently shown to be a competitive inhibitor of *Escherichia coli* agmatinase, with an inhibition constant (*K_i_*) of 1 mM, thus affecting agmatine catabolism in the intestine. Notably, metformin does not inhibit other ureohydrolase family homologs or enzymes that use agmatine as a substrate. Furthermore, structurally related biguanide compounds—phenformin, buformin, and galegin—displayed greater inhibitory potency against agmatinase, with *K_i_* values of 0.6, 0.1, and 0.007 mM, respectively [16].

**Figure 1:**
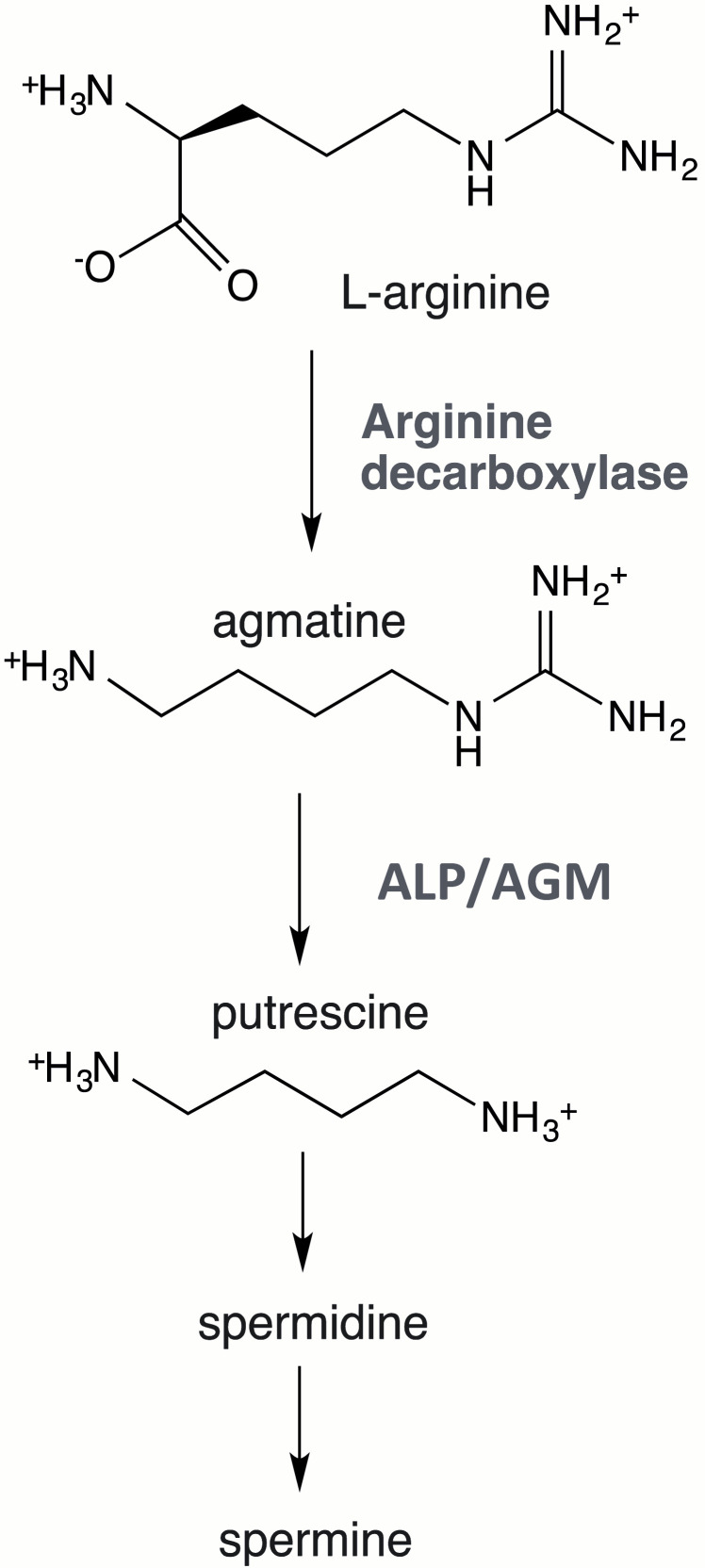
Pathways of agmatine biosynthesis and breakdown.

Agmatine catabolism varies across different organisms. In bacteria, high ADC and agmatinase (AGM) activities drive agmatine degradation (Figure 1), where agmatinase catalyzes its hydrolysis to putrescine and urea. In this context, putrescine serves as a precursor for polyamine biosynthesis (spermidine and spermine), which are essential for fundamental cellular processes such as transcription, translation, and DNA replication [17].

In plants such as *Arabidopsis thaliana*, agmatine also contributes to polyamine biosynthesis, although the specific pathways vary depending on tissue type and environmental factors [18]. In fungi, agmatine is catabolized via agmatine deiminase, yielding carbamoylputrescine and ammonia. Carbamoylputrescine then functions as a polyamine precursor [19]. In mammals, however, ornithine decarboxylase (ODC) is the key enzyme in polyamine synthesis, producing putrescine and CO_2_ from ornithine [20]. Consequently, agmatine is unlikely to act as a primary polyamine precursor in mammals and is instead proposed to function predominantly as a neuromodulator, interacting with multiple receptors at both central and peripheral levels [21].

In the case of human agmatinase (with orthologs in rats and mice), no agmatine hydrolytic activity has been conclusively demonstrated. Instead, this enzyme hydrolyzes other guanidine-containing substrates such as taurocyamine, guanidinobutyrate, and guanidinopropionate, but not agmatine [22]. These findings suggest that human agmatinase is not involved in the regulation of agmatine levels in mammals.

Several years ago, our laboratory identified a brain protein in rats that exhibits significant in vitro agmatinase activity [23]. The deduced amino acid sequence of this protein is highly divergent from all known ureohydrolases, lacking the conserved Mn^2+^-binding and catalytic residues characteristic of this enzyme family. We therefore referred to it as the agmatinase-like protein (ALP). ALP is expressed exclusively in mammals, with specific expression patterns in discrete brain regions of the rat [23]. Under in vitro conditions, ALP displays agmatinase activity with a *k_cat_* of 0.9 ± 0.2 s^−1^ and a *K_m_* of 3.0 ± 0.2 mM for agmatine [21,22]. Moreover, it supports polyamine biosynthesis from agmatine in *Saccharomyces cerevisiae* strains deficient in both ODC and ADC [23].

**Figure 2:**
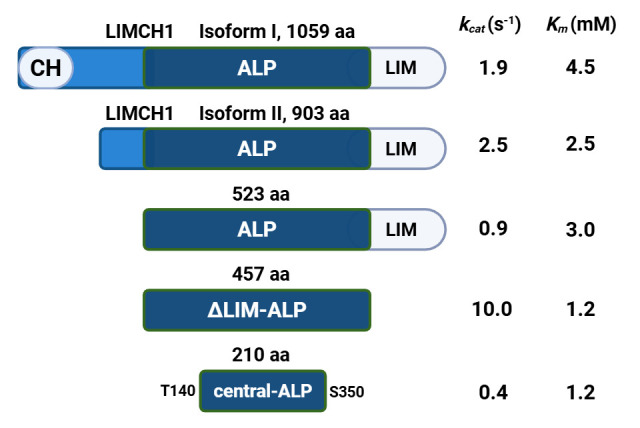
Comparative scheme of ALP and its variants. The regions shown in blue are identical between the different proteins [23,24,25].

According to database annotations, the ALP sequence (523 amino acids) of rat corresponds to the C-terminal extreme of the LIMCH1 protein (Figure S1). Our group has cloned and characterized several ALP variants, including the full-length LIMCH1 isoform (Figure 2). LIMCH1 isoforms 1 and 2 were cloned from rat hypothalamus mRNA [23], ALP was cloned from a rat brain cDNA library, ΔLIM ALP and central-ALP [24] were constructed in our laboratory. All variants exhibit agmatinase activity and require Mn^2+^ for catalysis, consistent with ureohydrolase enzymes. However, as noted, the ALP sequence lacks significant homology with known ureohydrolases and contains a LIM domain at its C-terminus.

A variant lacking this LIM domain, referred to as ΔLIM-ALP, demonstrates significantly enhanced catalytic activity compared to the full-length protein [25]. Specifically, ΔLIM-ALP shows a 10-fold increase in *k_cat_* and a 3-fold decrease in *K_m_* for agmatine [23,24]. This increase in activity upon deletion of a regulatory LIM domain has also been observed in unrelated proteins, indicating that such regulatory inhibition is not unique [26].

To date, no structural information is available for LIMCH1 or any ALP isoform, and protein structure prediction artificial intelligence algorithms such as AlphaFold have failed to generate accurate structural models (Figure 2). In this study, we purified two variants to homogeneity: full-length LIMCH1 (isoform I) and ΔLIM-ALP. We performed circular dichroism (CD) spectroscopy to gain insights into their secondary structure content. LIMCH1 was chosen due to its presumed physiological relevance as the full-length isoform, while ΔLIM-ALP was selected for its greater stability and higher enzymatic activity.

Furthermore, due to the absence of structural information, the location of the active site in ALP remains unknown. In a previous publication [24], several residues were proposed as potential ligands coordinating the Mn^2+^ cofactor—an essential component of ALP’s agmatinase activity. The candidate residues include E190, N213, Q215, D217, E288, K290, and N340. In this study, we generated single-point mutants of each of these residues and evaluated the impact of the substitutions on enzymatic activity and kinetics.

## 2. Materials and Methods

### 2.1. Materials

Agmatine, glycine, Tris, SDS, and all other reagents were of the highest quality commercially available (most from Sigma Aldrich Chemical Co., Louis, MO, USA). Restriction enzymes, as well as enzymes and reagents for PCR, are obtained from Invitrogen Co. (Carlsbad, CA, USA). The synthetic nucleotide primers were obtained from the Fermelo Biotec Co. (Santiago, Chile).

### 2.2. Bacterial Cultivation and Protein Overexpression and Purification

*E. coli* BL21 (λDE3) were grown in LB with 50 μg/mL ampicillin when transformed. ΔLIM-ALP and LIMCH1 gene cloned in pQE60 vector disposable in our laboratory [23] with an N-terminal 6xHis-tag. For recombinant protein expression, *E. coli* BL21 (λDE3) were transformed with the vector, grown in LB supplemented with 0.5 mM MnCl2, if not stated otherwise, at 37 °C to an OD600 of 0.6, transferred to 18 °C and induced over night with 1 mM IPTG. The cells were harvested by centrifugation, resuspended in lisis buffer (Tris-HCl 100 mM pH 7.5, MnCl_2_ 2 mM; putrescine 2 mM, PMSF 0.1 mM, DTT 5 mM y KCl 100 mM) and lysed by ultrasonication. After centrifugation at 12,000 g for 20 min at 4 °C, the soluble protein fraction was partially purified by mean of DEAE-cellulose anion exchange chromatography (calibrated with Tris-HCl 10 mM, pH 7, 5, MnCl2 0.2 mM), eluted with KCl 250 mM and an NTA–Ni^2+^ affinity chromatography (equilibrated with 2 mM imidazole and using 5 and 10 mM imidazole to elute in a gravity flow columns). Finally, a size-exclusion chromatography (HiLoad Superdex 200™ 200 pg 16/60, Cytiva, Marlborough, MA, USA) prep grade) was performed. For the expression and partial purification of the mutated variants, MnCl_2_ was excluded at all stages.

### 2.3. Circular Dichroism (CD) Measurement

CD measurements of delta-LIM ALP and LIMCH1 under different conditions were performed on a Jasco 1500 Plus spectropolarimeter (J-1500-150, JASCO Corp., Tokyo, Japan). In the experiment at different temperatures, the reaction mixture was maintained at the respective temperature throughout the experiment. All spectra were corrected by subtracting the buffer and buffer plus metal ion baselines recorded under the same conditions.

### 2.4. Mn^2+^ Determination in LIMCH1

The manganese content of LIMCH1 was determined by total reflection X-ray fluorescence analysis on a TXRF S4 Tstar Bruker spectrometer (Bruker Nano GmbH, Berlin, Germany). Sample preparation Stock solutions of 1000 mg L−1 of Ga (TraceCERT^®^, standard for ICP, Fluka^®^, Merck Sigma-Aldrich, Buchs, Switzerland) was used as an internal standard. The samples were prepared for their measurement by means of the drop drying method. This method consists of the deposition of a small volume of the mixture (in this case, 10-μL volumes) by means of a micropipette on the quartz sample disks of 30 mm diameter and drying by means of an infrared lamp.

Purified LIMCH1 was used at a concentration of 1.4 mg/mL and extensively dialyzed against 5 mM Tris-HCl pH 7.5 prior to analysis. The stoichiometry calculations were based on a previously determined subunit molecular mass of LIMCH1 (119.5 kDa).

### 2.5. Site-Directed Mutagenesis

The N213A, Q215A, D217A, E288A, K290A variants of ΔLIM-ALP were obtained by PCR, using the QuickChange site-directed mutagenesis kit (Stratagene, La Jolla, CA, USA); the plasmid pQE60 vector containing ΔLIM-ALP cDNA was used as template. The expected mutations were confirmed by DNA sequence analysis.

### 2.6. ΔLIM-ALP and LIMCH1 Activity Determination

Routinely, ALP activities were determined by measuring the formation of urea (product) using 80 mM agmatine in 50 mM glycine–NaOH (pH 9.0) and 5 mM MnCl2. All the assays were initiated by adding the enzyme to the substrate, buffer and MnCl2 solution were previously equilibrated at 37 °C, and urea was determined by the formation of a colored complex with α-isonitrosopropiophenone [23], measuring the absorbance at 540 nm. Initial velocity studies were performed in duplicate and repeated three times. Kinetic parameters were obtained by fitting the experimental data to the appropriate Michaellis–Menten equation (*v_i_* = *V_max_S*/*_Km_* + *S*) by using nonlinear regression with Graph Pad Prism version 7.0 for Windows (Graph Pad Software Inc., San Diego, CA, USA). Protein concentration was determined using the standard Bio-Rad protein assay (Bio-Rad, Hercules, CA, USA) with bovine serum albumin as standard.

### 2.7. Statistical Analysis

The results were evaluated with GraphPad Prism v7.0 using an analysis of variance (ANOVA), multiple comparison tests, or unpaired two-tailed *t*-test. All activity and kinetic parameter measurements were performed in at least three independent experiments, each in duplicate. Reported values correspond to the mean ± standard error of the mean (SEM). Statistical analyses were performed using GraphPad Prism v7.0, applying one-way ANOVA or Student’s *t*-test, as appropriate. 

## 3. Results

### 3.1. AlphaFold-Based Structural Predictions of LIMCH1 and ΔLIM-ALP

**Figure 3:**
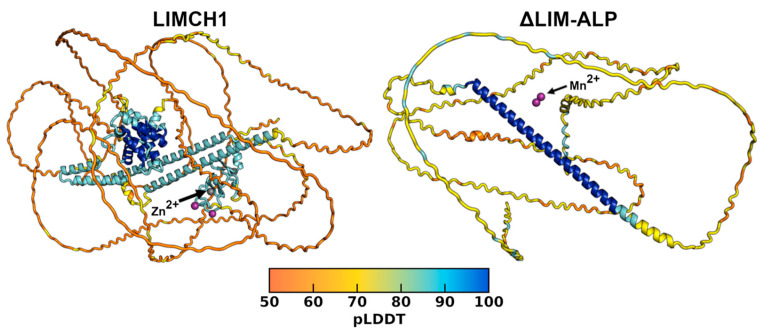
Structural models of LIMCH1 and ΔLIM-ALP predicted by AlphaFold3. The structures are colored according to the pLDDT score of each residue (scale below). In the LIMCH1 model, two Zn^2+^ ions (gray) and two Mn^2+^ ions (purple) are observed, while in the ΔLIM-ALP model only two Mn^2+^ ions (purple) are present.

Analysis of the LIMCH1 model predicted by AlphaFold2, available in the AlphaFold Database (code F1M392_RAT), revealed very low secondary structure content, with most of the protein predicted as loops and associated with low confidence scores. To evaluate the potential influence of Mn^2+^ and Zn^2+^ ions, we generated additional models using diffusion-based approaches in AlphaFold3. These models exhibited the same overall structural features as those obtained with AlphaFold2 (Figure 3). Across both algorithms, only a few segments were predicted with reasonable pLDDT scores. The Predicted Aligned Error (PAE) plots identified discrete regions with low error, corresponding to the calponin-like domain (residues 11–174), three α-helical regions (341–396, 411–457, 771–829), and the LIM domain (1005–1085). AlphaFold3 further predicted Zn^2+^ binding sites within the LIM domain, identifying the two canonical Zn^2+^-binding motifs characteristic of this domain (Figure 3). In contrast, Mn^2+^ binding sites were not reliably predicted, as the five generated models proposed different putative sites, none consistent with the coordination geometries typically observed for Mn^2+^. Analysis of the truncated ΔLIM-ALP predicted even fewer structured regions, with only a single central helix showing low PAE values and no reliable binding sites for the two Mn^2+^ ions (Figure 3).

### 3.2. Purification and Circular Dichroism Analysis of LIMCH1 and ΔLIM-ALP

Using ion-exchange chromatography (DEAE-cellulose Figure S2), His-tag affinity chromatography (Ni^2+^-NTA agarose), and size-exclusion chromatography (Superdex 200™ prep grade), we purified LIMCH1 and ΔLIM-ALP (Figure S3) to a level of homogeneity suitable for circular dichroism (CD) analysis.

**Figure 4:**
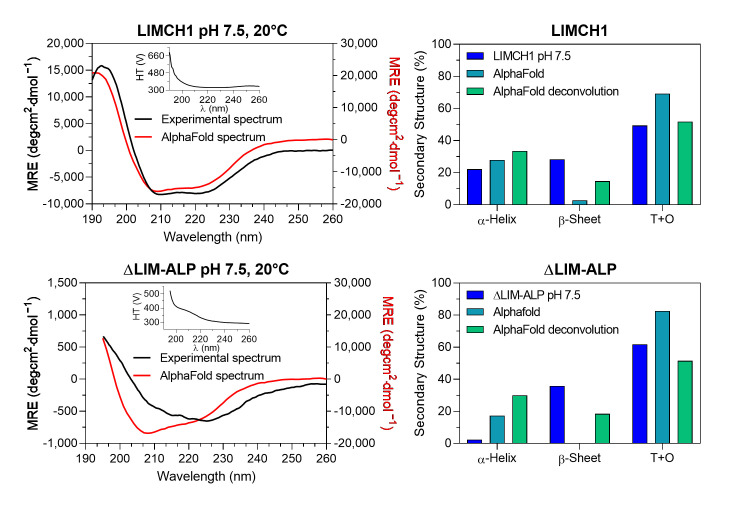
Secondary structure analysis comparing experimental circular dichroism (CD) spectra at pH 7.5 with theoretical spectra derived from AlphaFold3 models of LIMCH1 and ΔLIM-ALP. **Upper panel** (LIMCH1, 0.16 mg/mL): **On the left**, the experimental CD spectrum at pH 7.5 and 20 °C (black line, left Y-axis) is compared with the theoretical spectrum calculated from the AlphaFold3 model (red line, right Y-axis). The dichroic signal is expressed as Mean Residue Ellipticity (MRE). The inset shows the high-tension (HT) voltage recorded during measurement. **On the right**, secondary structure content is presented as follows: LIMCH1, percentage of secondary structure determined by deconvolution of the experimental CD spectrum with BeStSel; AlphaFold, percentage of secondary structure predicted directly from AlphaFold3 structural models; AlphaFold deconvolution, percentage of secondary structure obtained from the theoretical CD spectrum derived from the AlphaFold3 model, deconvoluted with BeStSel (T+O, for Turns and Other). **Lower panel** (ΔLIM-ALP, 0.91 mg/mL): The same analyses are shown under identical experimental and pH conditions.

The experimental CD spectra of LIMCH1 exhibited approximately half the signal intensity at 222 and 208 nm compared to the theoretical spectra predicted from the AlphaFold structural models (Figure 4). In the case of ΔLIM-ALP, the CD signal was nearly tenfold weaker than theoretically expected, making accurate CD measurements technically challenging due to the low signal-to-noise ratio (Figure 4). This reduced signal suggests that these proteins may contain even fewer structured regions than predicted, behaving largely as intrinsically disordered proteins, particularly in the case of ΔLIM-ALP.

Deconvolution of the experimental LIMCH1 spectrum using BeStSel indicated that loops and turns (T+O) account for 49% of the structure, whereas the AlphaFold model predicts 69%. This discrepancy may reflect BeStSel’s bias toward compact globular proteins, where β-sheet content is often overestimated. For example, deconvolution of the theoretical spectrum generated from the AlphaFold model yields 14% β-sheets, while the structural model itself contains only 3%, resulting in an underestimated proportion of disordered regions. Nevertheless, this approach indicates that at least 50% of LIMCH1 is intrinsically disordered, consistent with AlphaFold predictions.

For ΔLIM-ALP, deconvolution of the experimental spectrum indicated 62% or more disordered structures (T+O). As observed for LIMCH1, the β-sheet content appears overestimated, with BeStSel assigning 18% despite the AlphaFold model lacking β-sheets. These results support the conclusion that ΔLIM-ALP is even more disordered than LIMCH1, in agreement with AlphaFold predictions. It is important to note that the models generated by AlphaFold represent static conformations and do not capture conformational heterogeneity or dynamic disorder. In contrast, circular dichroism reflects the average structural content of a dynamic ensemble of conformations in solution. Therefore, the observed differences reflect the intrinsic flexibility of LIMCH1 and ΔLIM-ALP, which cannot be represented by a static model.

These structural characteristics of LIMCH1 and ΔLIM-ALP differ markedly from those observed in classical ureohydrolases. For instance, the monomeric form of *E. coli* agmatinase (EcAGM) possesses a well-defined α/β/α sandwich fold, comprising a central eight-stranded parallel β-sheet flanked by ten α-helices and five 3_10_ helices [27]. Its secondary structure composition includes 46.9% loop and turns regions, 13.6% β-structures, 33.3% α-helices, and 6.1% 3_10_ helices. While EcAGM also contains a notable proportion of T+O elements, its higher helical content stands in clear contrast to the structural profiles of both LIMCH1 and ΔLIM-ALP. In addition, the *E. coli* agmatinase has a *K_m_* for its substrate agmatine of 1.1 mM and a catalytic constant *k_cat_* of 140 s^−1^ [27].

### 3.3. Thermal Hyperactivation of LIMCH1 and ΔLIM-ALP

**Figure 5:**
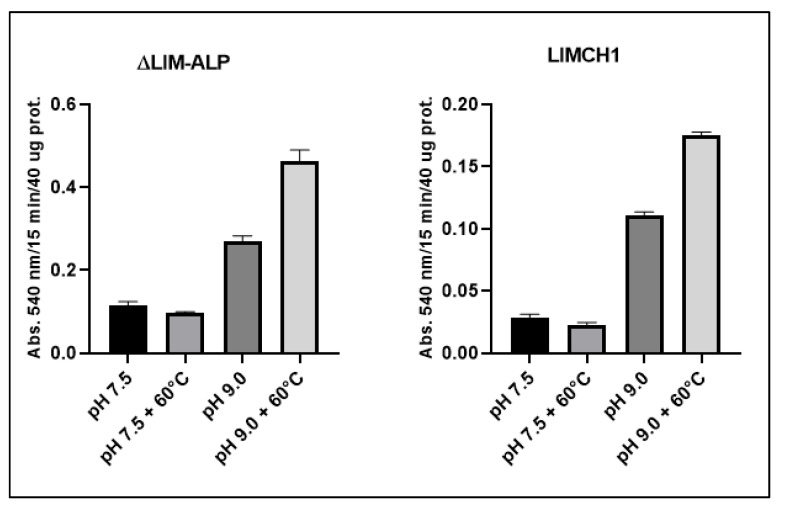
Agmatinase activity of ΔLIM-ALP and LIMCH1 measured in Tris-HCL 50 mM pH 7.5 and in Glicine-NaOH 50 mM pH 9.0, with agmatine 20 Mm and MnCl_2_ 2 mM. For hyperactivation assays, samples were incubated with 5 mM MnCl_2_ at 60 °C for 5 min, cooled to room temperature, and enzymatic activity was subsequently measured at 37 °C under the indicated pH conditions. For this experiment, the enzymes were partially purified by DEAE-cellulose chromatography.

As shown in Figure 5, the activity of both LIMCH1 and ΔLIM-ALP more than doubles at pH 9.0. Additionally, enzymatic activity is significantly enhanced following incubation at 60 °C in the presence of Mn^2+^, followed by cooling and measurement—an effect known as hyperactivation, which is characteristic of ureohydrolases [28]. Notably, this hyperactivation is only observed at alkaline pH (pH 9.0). In contrast, incubation in the absence of Mn^2+^ results in complete loss of activity in both protein variants [28].

**Figure 6:**
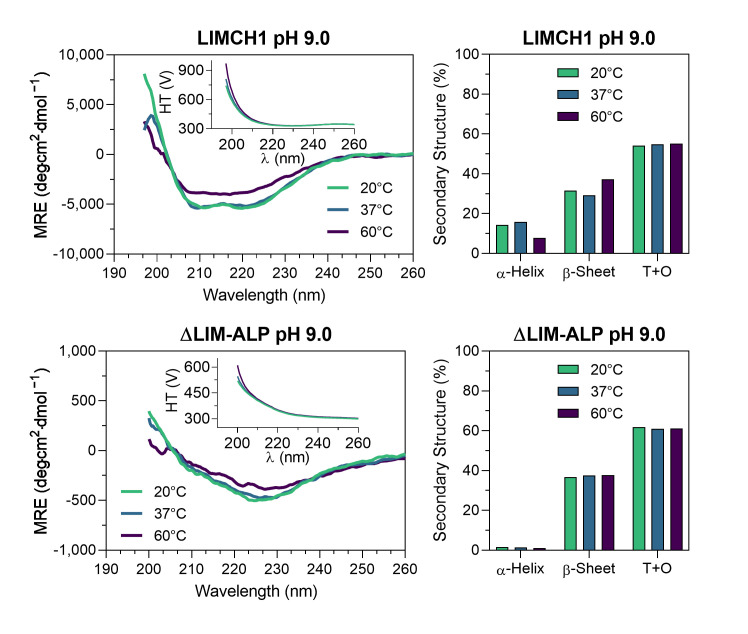
**Thermal stability analysis by CD of LIMCH1 and ΔLIM-ALP at pH 9.0.**  The  **upper panel** shows the data for LIMCH1 (0.16 mg/mL) at pH 9.0. **On the left**, the CD spectra measured at 20 °C, 37 °C, and 60 °C are presented, with an inset displaying the high-tension (HT) voltage signal recorded during measurements at each temperature. **On the right**, the secondary structure deconvolution of the CD spectra at different temperatures is shown, indicating the percentage of α-helices, β-sheets, and the sum of turns and disordered structures (T+O, for Turns and Other). In the **lower panel**, the same analyses are shown for ΔLIM-ALP (0.91 mg/mL) under the same experimental and pH conditions.

Figure 6 shows the effect of temperature on the secondary structure content of the protein variants at pH 9.0. The analysis was performed at room temperature (20 °C), physiological temperature (37 °C)—which corresponds to the temperature used in enzymatic activity assays—and at 60 °C. Minimal changes in secondary structure were observed, and only for LIMCH1: as temperature increased, its α-helical content decreased slightly while β-sheet content increased modestly, without accounting for potential biases of BeStSel with these proteins. Nevertheless, the spectra at 60 °C indicate that these proteins maintain their overall structure, raising the intriguing question of how proteins with such a high proportion of disordered regions can exhibit catalytic activity and phenomena such as hyperactivation, classically observed in canonical ureohydrolases [28].

### 3.4. Site-Directed Mutagenesis of Putative Mn^2+^-Binding Ligands in ALP

In a previous publication we reported that dialyzed samples of ΔLIM-ALP contain one Mn^2+^ ion per protein monomer [25]. In the present study, the Mn^2+^ content of the LIMCH1 protein was determined using total reflection X-ray fluorescence (TXRF) analysis, revealing that LIMCH1 contains 1,3 Mn^2+^ ion(s) per subunit.

Given the essential role of the Mn^2+^ cofactor in the catalytic activity of ureohydrolases, we proposed several residues as potential Mn^2+^-coordinating ligands within the active site of ALP. These residues were selected based on a previously published structural model [26], which was constructed despite the low sequence identity between ΔLIM-ALP and prokaryotic agmatinases. This study aimed to provide insight into the possible location and architecture of the ALP active site.

We generated five single-point mutants targeting the proposed Mn^2+^-binding residues: N213A, Q215A, D217A, E288A, and K290A. To avoid any interference with potential metal coordination, Mn^2+^ was excluded from the expression and all purification steps, and the proteins were not subjected to thermal activation at 60 °C in the presence of Mn^2+^. Each variant was partially purified using DEAE-cellulose chromatography (10 mM Tris-HCl, pH 7.5) and eluted with 250 mM KCl.

**Figure 7:**
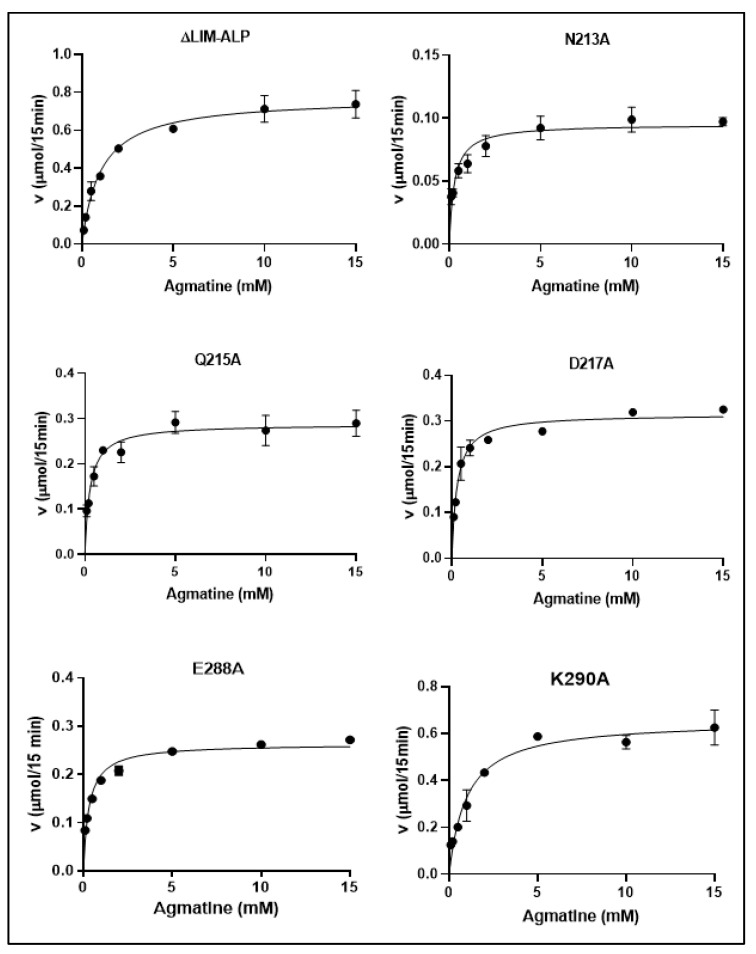
Saturation curve at known concentrations of agmatine. for N213A, Q215A, D217A, E288A, K290A and ΔLIM-ALP. The GraphPad Prism 8 program was used, and nonlinear adjustments were used.

Kinetic analyses (Figure 7) revealed that all mutant enzymes followed Michaelis–Menten kinetics, displaying hyperbolic substrate saturation curves for agmatine, as analyzed using GraphPad Prism 8. Interestingly, four of the five mutants exhibited a significant decrease in *K_m_* values compared to the wild-type ΔLIM-ALP enzyme (Table 1). While the wild-type enzyme displayed a *K_m_* of approximately 1.0 mM, the N213A, Q215A, D217A, and E288A mutants showed *K_m_* values of ~0.3 mM, indicating an approximate threefold increase in substrate affinity. These findings suggest that the individual mutations may enhance enzyme–substrate interactions.

In contrast, the *V_max_* normalized to total protein concentration was reduced in all five variants relative to the wild-type enzyme. Notably, the catalytic efficiency (*V_max_*/*K_m_*) was significantly altered only in the N213A, E288A, and K290A mutants, indicating that these residues may play a more direct role in the coordination of Mn^2+^ or in maintaining the structural integrity of the active site.

The observation that the point mutations significantly altered the kinetic parameters is particularly noteworthy, as it suggests that these residues could be involved in or near the active site of ΔLIM-ALP. To analyze this aspect, a molecular dynamics simulation was performed to allow for protein stabilization in each of the mutants. The structures of the corresponding mutations showed no structural changes (drms < 1.0 Å), indicating that the changes in enzymatic activity were not due to major conformational changes in the protein.

The observation that point mutations significantly alter the kinetic parameters is particularly noteworthy, as it suggests that these residues are directly involved in or in close proximity to the active site of ΔLIM-ALP.

To further assess the impact of the mutations on Mn^2+^ coordination, enzymatic activity of the partially purified variants was measured in the presence and absence of Mn^2+^. In addition, the samples were dialyzed against 5 mM Tris-HCl (pH 7.5) to remove residual metal ions, followed by activity assays with and without Mn^2+^ supplementation.

**Figure 8:**
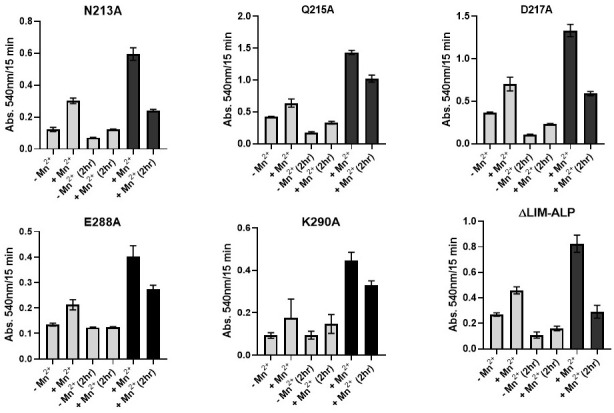
Effect of Mn^2+^, dialysis and hyperactivation on the activity of wild-type and mutant enzymes of ΔLIM-ALP. Light bars correspond to non-hyperactivated enzymes, measured with and without Mn^2+^ before and after dialyzing. Dark bars indicate the activity of hyperactive enzymes with Mn^2+^ before and after dialysis for 2 h against 5 mM Tris-HCl pH 7.5. The activity is graphed without normalizing by protein concentration.

Hyperactivation assays were also conducted by incubating the proteins at 60 °C, both in the presence and absence of Mn^2+^, followed by activity measurements under both metal-supplemented and metal-free conditions. The results of these experiments are summarized in Figure 8.

As shown in Figure 8, all variants exhibited agmatinase activity both in the presence and absence of added Mn^2+^. Upon addition of Mn^2+^, activity increased by 30–50%, and no significant differences were observed compared to the wild-type enzyme. After subjecting the variants to a 2 h dialysis, all lost enzymatic activity, including the wild-type. In the case of variants E288A and K290, the dialyzed enzymes displayed activity levels comparable to those of the untreated enzymes measured in the absence of Mn^2+^. Upon Mn^2+^ addition, only partial recovery of activity was observed in all dialyzed samples.

In the hyperactivation assay at 60 °C with Mn^2+^, nearly all variants showed a two-fold increase in activity. Following dialysis, activity decreased by approximately 30–50%.

For ureohydrolases such as arginases and *E. coli* agmatinase [28], the presence of a tightly bound Mn^2+^ ion—retained after dialysis—and a more loosely bound Mn^2+^ ion—lost during dialysis and associated with activity loss—has been reported. A similar behavior was observed in ΔLIM-ALP despite lacking the classical residues involved in metal ion coordination in ureohydrolases. No substantial differences in behavior were observed between the mutated variants and the wild-type enzyme.

## 4. Discussion

This study provides compelling evidence that LIMCH1 and its C-terminal variant, ΔLIM-ALP, represent a rare example of protein-mediated enzyme catalysis with substantial intrinsic disorder. Structural predictions using AlphaFold3 generated low-confidence models dominated by unstructured regions, particularly in ΔLIM-ALP. This outcome may reflect limitations of current AI-based prediction tools or the possibility that LIMCH1 is inherently unstructured.

In contrast, circular dichroism (CD) analysis indicated that LIMCH1 contains significantly more secondary structure than predicted, including defined β-sheets and α-helices. However, this method may introduce bias, as deconvolution algorithms are optimized for highly structured proteins. Both LIMCH1 and ΔLIM-ALP exhibit robust agmatinase activity, display Mn^2+^ dependence, and undergo Mn^2+^-dependent thermal hyperactivation—hallmarks of canonical ureohydrolases. Nonetheless, their secondary structure profiles appear distinct, lacking the classical α/β/α sandwich fold according to AlphaFold predictions. ΔLIM-ALP, in particular, appears to be even more structurally disordered than LIMCH1. Despite this, it retains catalytic activity—an unusual finding, as enzymatic function is generally associated with a well-ordered tertiary structure.

Taken together, our results demonstrate that LIMCH1 and ΔLIM-ALP retain functional enzymatic activity despite being largely disordered—an uncommon and underexplored phenomenon in enzymology [29]. Recent research shows that while many enzymes require a specific structure, some proteins with disordered regions use flexibility and dynamics to catalyze reactions, such as the protein UREG, which requires a “window of flexibility” to function, or a recently discovered disordered domain that catalyzes the polymerization and depolymerization of microtubules [30,31].

Interestingly, thermal hyperactivation was observed only at pH 9.0, and only minor structural changes were detected upon heat treatment. LIMCH1 showed a slight decrease in α-helical content at elevated temperatures, while ΔLIM-ALP showed no detectable alterations despite a comparable increase in enzymatic activity. This suggests that activation may involve subtle or localized rearrangements not captured by global secondary structure analysis. Collectively, these findings reinforce the idea that LIMCH1 is not a well-folded protein and highlight the challenge of understanding how a largely unstructured protein can exhibit the kinetic features of canonical ureohydrolases.

Site-directed mutagenesis of five putative Mn^2+^-binding residues in ΔLIM-ALP (N213A, Q215A, D217A, E288A, and K290A) revealed that none abolished agmatinase activity, indicating that these residues are not essential for metal coordination. However, most mutants showed reduced *K_m_* values, suggesting an optimized interaction with the substrate agmatine, possibly due to changes in the local environment of a putative active site. All variants displayed decreased *V_max_*, and three (N213A, E288A, K290A) showed altered catalytic efficiency (*V_max_*/*K_m_*), indicating that these residues contribute to modulating substrate processing or maintaining a putative active site architecture. Furthermore, the Mn^2+^-dependent activation profiles and recovery upon dialysis were similar between mutant and wild-type enzymes, suggesting that Mn^2+^ binding capacity is largely conserved. These results indicate that the mutated residues influence substrate interaction and catalytic turnover but are not directly responsible for coordinating the activating Mn^2+^ ion.

## 5. Conclusions

This study establishes LIMCH1 and its C-terminal variant ΔLIM-ALP as rare examples of catalytically competent disordered enzymes. Despite extensive structural disorder predicted by state-of-the-art modeling tools (AlphaFold3), both proteins display robust Mn^2+^-dependent agmatinase activity and thermal hyperactivation—features characteristic of canonical ureohydrolases. The discrepancy between computational models and spectroscopic data underscores both the limitations of current structure-prediction algorithms for disordered proteins and the unusual structural adaptability of LIMCH1.

Point mutations in ΔLIM-ALP significantly affected enzyme kinetics, implying that the substituted residues are located within or near the catalytic region. The Mn^2+^-dependent activity patterns observed between the mutants and the wild-type enzyme, along with the partial recovery of activity after dialysis, suggest the presence of both strongly and weakly bound Mn^2+^ ions, as described for canonical ureohydrolases. Surprisingly, ΔLIM-ALP exhibits this double metal behavior despite lacking the conserved residues typically required for metal coordination, revealing a non-canonical Mn^2+^ bonding mechanism.

The preservation of agmatinase activity in the absence of canonical structural motifs and classical metal-coordinating residues suggests that LIMCH1 may have undergone evolutionary selection for this function, representing a striking case of convergent evolution with canonical ureohydrolases. This work broadens our understanding of the structural plasticity compatible with enzyme catalysis and raises important questions about the evolutionary mechanisms that enable catalysis in intrinsically disordered proteins.

## Figures and Tables

**Table 1:** Binding site in ΔLIM-ALP.

	*K_m_* (mM)	*V_max_* (Abs_540nm_/15 min/75 μg Proteins)	*V_max_*/*K_m_*
ΔLIM-ALP	1.04 ± 0.10	1.55 ± 0.04	1.49
N213A	0.34 ± 0.09	0.19 ± 0.01	0.55
Q215A	0.29 ± 0.04	0.44 ± 0.01	1.51
D217A	0.29 ± 0.03	0.56 ± 0.01	1.93
E288A	0.32 ± 0.03	0.26 ± 0.01	0.8
K290A	1.00 ± 0.16	0.32 ± 0.03	0.32

## Data Availability

The original contributions presented in this study are included in the article/Supplementary Materials. Further inquiries can be directed to the corresponding author.

## Supplementary Materials

The following supporting information can be downloaded at: https://www.mdpi.com/article/10.3390/biom15111620/s1, Figure S1. Alignment between LIMCH1 isoforms 1 and 2 and ALP; Figure S2. DEAE-cellulose gravity chromatography (10 mM Tris-HCl, pH 7.5) of ΔLIM-ALP; Figure S3. Purification results of LIMCH1 (left) and ΔLIM-ALP (right) after the 3 chroma-tographies: DEAE-cellulose, His-tag affinity chromatography (Ni^2+^-NTA agarose), and size-exclusion chromatography (Superdex 200™ prep grade).
