# Supplementary material for: Structural and Functional Characterization of LIMCH1 and Its Agmatinase-like Region: A Case of Catalysis in a Highly Disordered Protein
Source: Biomolecules. 2025 Nov 18;15(11):1620. doi: 10.3390/biom15111620 (PMC12650571; doi:10.3390/biom15111620)
Supplement: Supplementary file 1 [file biomolecules-15-01620-s001.zip › biomolecules-3931499-supplementary.pdf]

Supplementary Materials

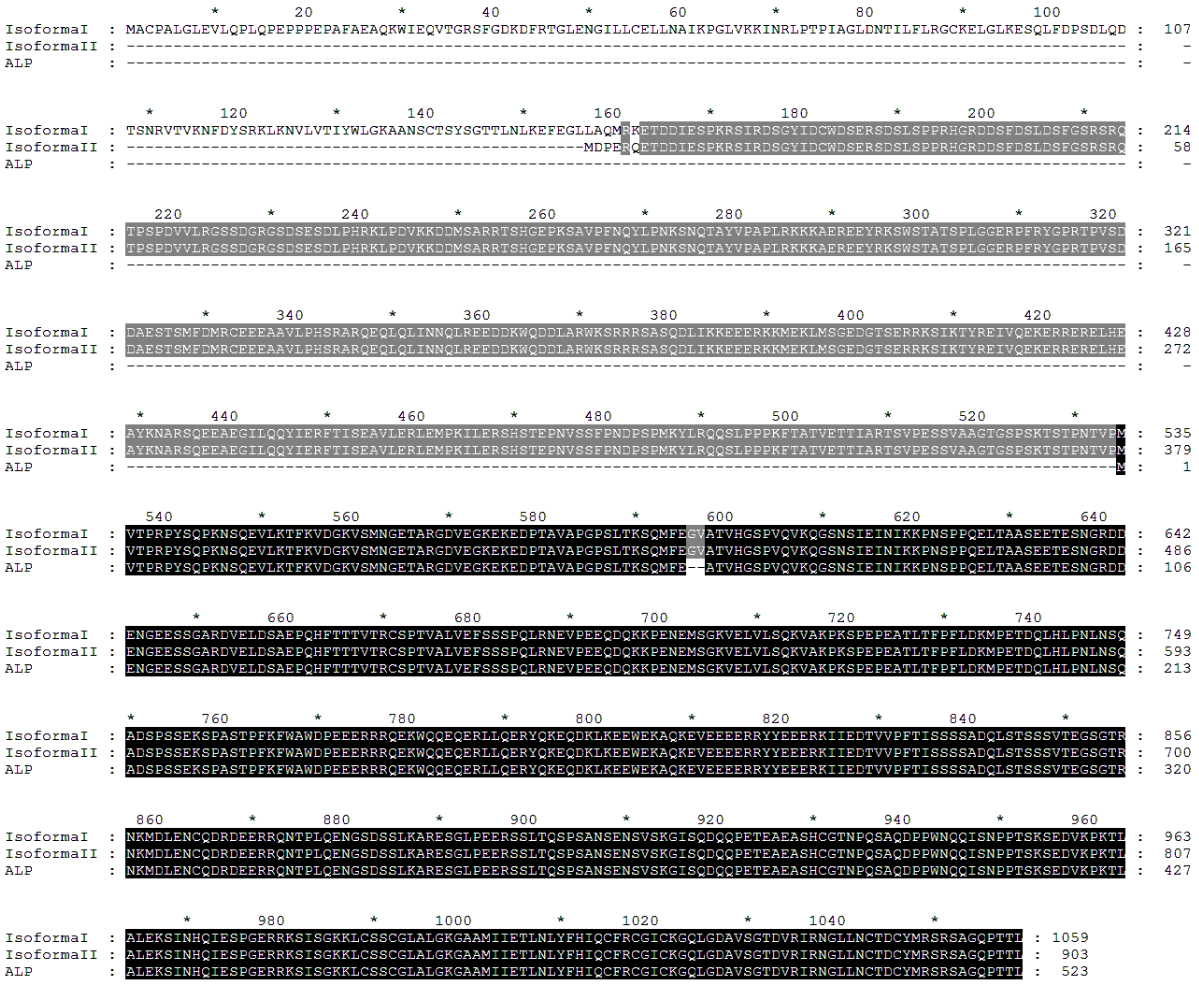

Figure S1. Alignment between LIMCH1 isoforms 1 and 2 and ALP.

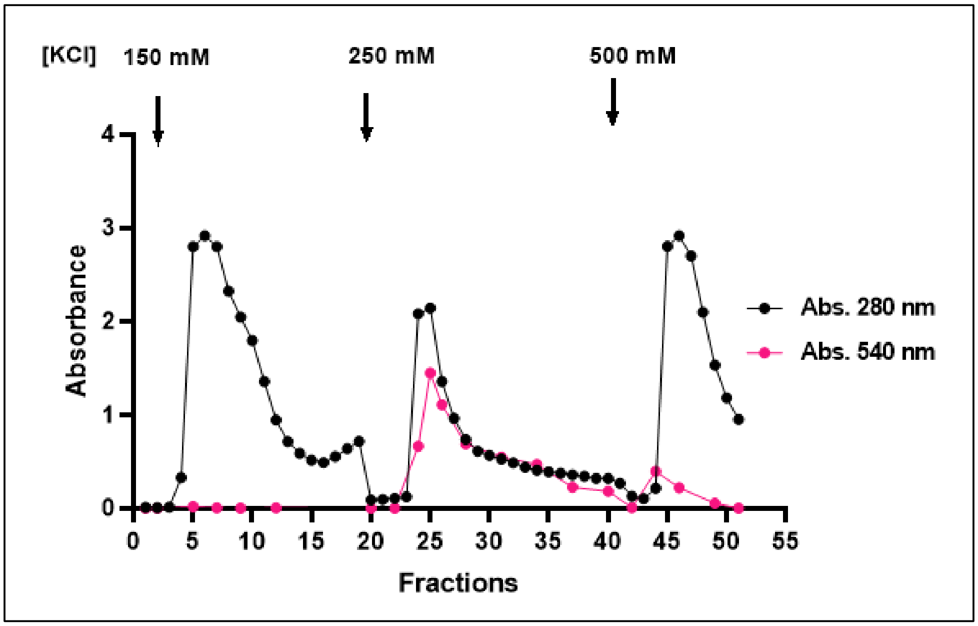

Figure S2. DEAE-cellulose gravity chromatography (10 mM Tris-HCl, pH 7.5) of ΔLIM-ALP. The protein extract was chromatographed and the proteins were eluted with a discontinuous KCl gradient. ΔLIM-ALP was eluted with 250 mM KCl and *E. coli* agmatinase was eluted with 500 mM KCl. Equivalent results were obtained with LIMCH1. Absorbance at 280 indicates total protein content and absorbance at 540 indicates agmatinase activity.

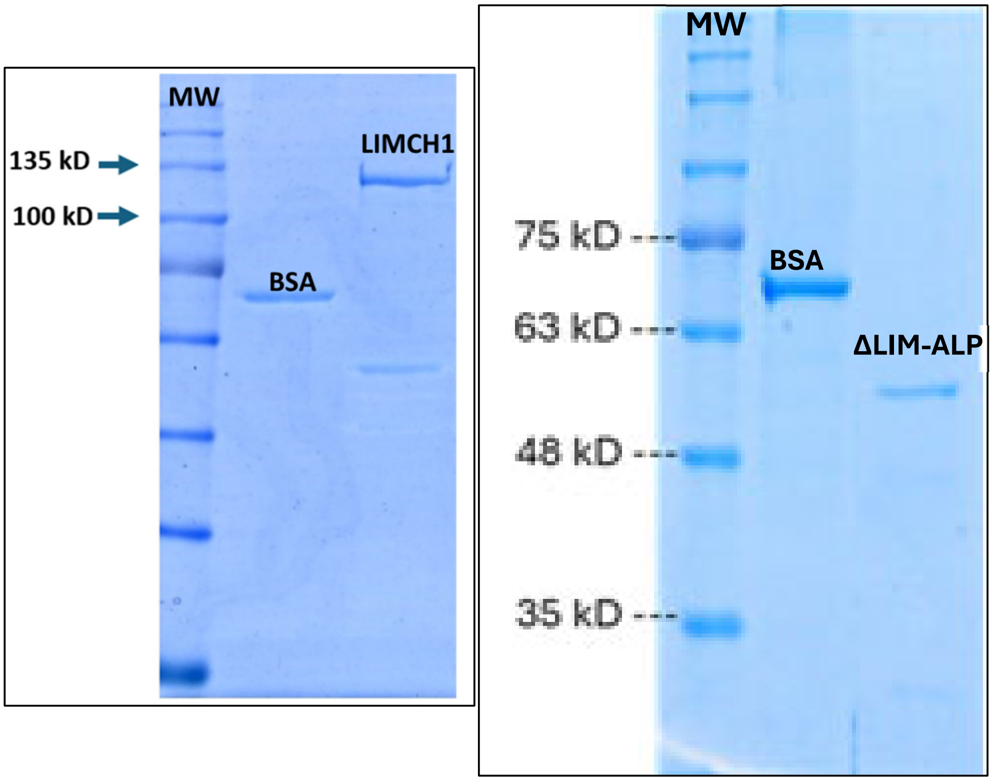

Figure S3. Purification results of LIMCH1 (left) and ΔLIM-ALP (right) after the 3 chromatographies: DEAE-cellulose, His-tag affinity chromatography (Ni^{2+}-NTA agarose), and size-exclusion chromatography (Superdex 200™ prep grade). MW: Molecular weight marker, BSA: Bovine serum albumin.
